# Green and simple microextraction method based on deep eutectic solvent-assisted smartphone digital imaging colorimetry (SDIC) for Rhodamine B analysis in food products

**DOI:** 10.1007/s00216-025-05932-x

**Published:** 2025-06-05

**Authors:** Buse Tezcan, Aslıhan Dalmaz, Sezen Sivrikaya Özak

**Affiliations:** 1 Department of Chemistry, Graduate Education Institute, Düzce University, Düzce, 81620 Türkiye; 2 Department of Chemistry, Faculty of Art and Science, Düzce University, Düzce, 81620 Türkiye

**Keywords:** Smartphone digital image colorimetry (SDIC), Deep eutectic solvent, Green chemistry, Rhodamine B, Food samples

## Abstract

This study employed a deep eutectic solvent-based liquid-liquid microextraction method and smartphone digital image colorimetry to detect and quantify Rhodamine B in foods. The deep eutectic solvent, a green solvent, was prepared by mixing tetrabutylammonium bromide and octanol in a 1:2 ratio. It was observed that Rhodamine B dyestuff readily transitioned to the DES phase, eliminating the need for additional reagents. The impact of various parameters, including DES type, molar ratio, volume, solution pH, ultrasonic time, centrifugation time, and sample volume, on extraction efficiency was examined, and the most effective conditions were determined. Additionally, the diameter of the prepared colorimetric box and the distance between the detector and the sample were investigated, leading to the identification of optimal conditions. The developed method’s limit of detection (LOD) and limit of quantification (LOQ) were found to be 0.0054 μg/mL and 0.018 μg/mL, respectively, for the measurements taken using seven parallel blank solutions at 554 nm. The relative standard deviations intraday and interday were 1.29% and 2.03%, respectively. The calibration graph demonstrated good linearity, with a coefficient of determination of 0.9996. The developed method’s intraday and interday relative standard deviation values were 2.03% and 1.29%, respectively. Rhodamine B was detected in food samples using the developed method, obtaining extraction recoveries ranging from 95.16 to 103.88%.

**Graphical Abstract:**

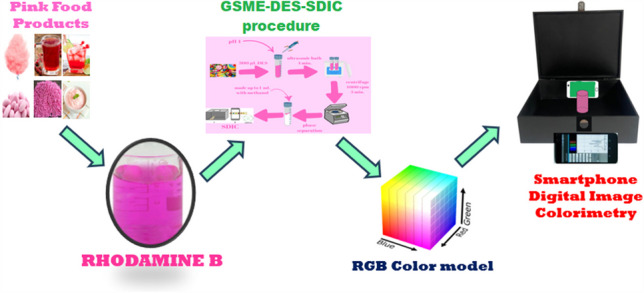

## Introduction

Dyes are frequently used in various industries, including textiles and food processing [1]. These colorants have the potential to cause harmful effects on human health and the environment [2]. Rhodamine B, a bright red fluorescent dye, is frequently used in research studies. The disadvantages associated with traditional methods have led to the development of contemporary approaches. The food industry uses a range of dyes, including fluorescent and toxic varieties, that pose potential risks to public health [3, 4]. Rhodamine B, used in various industries, including paper, paint, textile, and plastics, is particularly valuable in scientific research due to its fluorescent properties. It is essential to note that Rhodamine B irritates the skin, eyes, and respiratory system and is dangerous if swallowed [5]. Although many countries have banned its use in the food and pharmaceutical industries due to its toxicity, this dye remains in laboratory settings. In 1987, the International Agency for Research on Cancer classified Rhodamine B as a Group 3 carcinogen [6, 7]. Furthermore, Rhodamine B dye has been shown to cause chronic toxicity in animal models. As a result, accurate and reliable detection of Rhodamine B in food products, as in many samples, is of great importance [8, 9].

A significant proportion of research in analytical chemistry utilizes traditional methodologies. Despite their crucial role in research, the disadvantages of these approaches (such as maintenance costs [10–12], lengthy investigations, and dependence on laboratory environments) have prompted researchers to develop alternative techniques. The advent of advanced technology has stimulated the development of an unconventional method known as smartphone digital image colorimetry (SDIC), which aims to provide a high degree of flexibility for fast, cost-effective, and in situ analysis [13–15].

Smartphone digital image colorimetry is an analytical method that uses color changes to determine the concentration of a substance. The technique uses a smartphone camera to measure and analyze color intensities, thus providing a cost-effective and portable alternative, especially in scenarios where access to laboratory facilities is restricted [16–19]. The technique involves examining the color of a sample using photographs taken with a smartphone camera [20, 21]. The sample concentration is then determined by image analysis software that measures the intensities in RGB (red-green-blue) color channels [22]. A calibration graph can be created using absorbance or reflectance to quantify the amount of analyte in the sample. While previous researchers preferred continuous beam sources to illuminate the sample in a colorimetric box [23, 24], recent observations have shown that switching from continuous beam sources to a monochromatic source increases the sensitivity, selectivity, and linear dynamic range of SDIC, thus extending its applicability to the analysis of complex structures such as food [25, 26]. The accuracy of SDIC research results depends on several factors, including proper calibration, rigorous sample analysis, precise wavelength determination, and correct interpretation of the obtained data by the study’s investigator.

The choice of deep eutectic solvents (DES) as sample preparation media is based on the premise that they exhibit lower environmental impact than conventional solvents. These solvents offer a range of environmentally friendly properties compared to traditional solvents and thus serve as an alternative for applying green chemicals [27–30]. These compounds have found wide application in analytical chemistry in extraction and separation processes [31–35]. This alternative method is frequently used to extract food, pharmaceuticals, dyes, and pesticides [36–39]. In addition, DESs are used as electrolytes in electrochemical applications. Their low toxicity has made them popular in the pharmaceutical and cosmetic industries. The composition of DES typically consists of a hydrogen bond acceptor (HBA) and a hydrogen bond donor (HBD), which combine to form a new mixture with a melting point lower than their melting point [40–42]. The preference for these solvents can be attributed to their low cost, environmental safety, biodegradability, and tunable properties such as melting point and viscosity.

Many spectrophotometric and chromatographic methods are used to determine the targeted analytes [43]. Some of the most frequently preferred methods in the literature are those using ultraviolet-visible spectroscopy (UV-Vis), liquid chromatography (LC), and high-performance liquid chromatography (HPLC) analysis techniques [44–46]. Recently, with the introduction of the SDIC method into the literature, the analysis process has become more practical, and it is more advantageous in terms of accessibility. Compared to studies involving HPLC, LC, and UV-Vis methods, the SDIC method using smartphone cameras eliminates the need for both expensive and complex instrumentation. In another aspect, it is necessary to have devices such as ultrasonic/vortex and centrifuge to conduct a similar study. Although these devices are needed for the application of this method outside the laboratory, when evaluated in general, the advantages of the method bring SDIC analysis to the forefront and increase its preferability. The SDIC method facilitates access to analytical methods in areas where access to resources is limited and also provides rapid implementation. In addition, it not only helps to reduce costs but is also a portable and practical method. Moreover, with the development of technology, the advanced processing capabilities of smartphone cameras have increased the reliability and accuracy of the SDIC method. These advantages of the SDIC method suggest that it could be a potential alternative to traditional methods. Along with these methods, supramolecular solvent microextraction [47, 48], solid-phase microextraction [49, 50], and deep eutectic solvent microextraction procedures [51–53], which have selectivity, high sensitivity, and low detection limits, are applied. Researchers use these methods in most of their studies to obtain better results.

This study detected and quantified Rhodamine B in foods using a deep eutectic solvent-based liquid-liquid microextraction method coupled with smartphone digital image colorimetry. The aim was to develop a deep eutectic solvent liquid-liquid microextraction method coupled with smartphone digital image colorimetry to facilitate rapid and sensitive color change analysis and thus develop more reliable and cost-effective food analysis techniques. A colorimetric box was used in the laboratory to hold sample solutions, and the sample solution images were divided into red, green, and blue channels. The intensity of the green channel was used to calculate the dye concentration. Various parameters were studied, and the steps of the method were determined to ensure optimum conditions. The study is both a first and new in analyzing Rhodamine B in different food samples by deep eutectic solvent microextraction coupled with the SDIC method.

## Material and methods

### Reagents and chemicals

All chemicals and reagents used in this study are of standard quality and do not require purification. The Milli-Q system (Millipore, Billerica, MA, USA) was preferred for deionized water (DW) used to prepare sample solutions. Tetrabutylammonium bromide, menthol, thymol, coumarin, octanol, and decanoic acid chemicals were supplied by Merck (Darmstadt, Germany). Rhodamine B dye was selected as the standard material, and Merck provided the dye (Darmstadt, Germany). In addition, Sigma-Aldrich supplied sodium hydroxide and hydrochloric acid chemicals used during pH adjustment (Sigma-Aldrich, St. Louis, MO, USA).

### Instrumentations

The features of the phone selected to be used as a detector are as follows: The features of the phone are as follows: 6.2-inch screen, 421 ppi pixel density, 1080 × 2400 resolution, 8 GB RAM, 128 GB storage, 10 MP front camera, 12 MP + 12 MP + 64 MP rear camera. The features of the iPhone 6 brand phone used as a light source are 750 × 1334 pixels resolution, 4.7-inch screen, 1 GB RAM, 32 GB internal memory, 8 MP rear camera, and 1.2 MP front camera. The screenshot of the color corresponding to the 554-nanometer wavelength, the wavelength at which Rhodamine B dye shows maximum absorption, was obtained from a complementary application and transferred to the phone mentioned above, used as a light source.

A precision balance was used to weigh the samples accurately (Weightlab Instruments, Istanbul, Türkiye). A Hydraultrasonic brand UltraGold model ultrasonic bath (HYDRA Ultrasonic, Istanbul, Türkiye) was used to increase the interaction between DES and the analyte, and a VWR brand HIMAC CT GEL model centrifuge (VWR International GmbH, Darmstadt, Germany) was used to provide a clear phase separation. A PerkinElmer Frontier Optica Fourier Transform Infrared (FT-IR) spectrometer was used to identify the functional groups present in the structure of the DES compound and its constituent HBA and HBD components and to determine the formation of hydrogen bonds. The ^1^H nuclear magnetic resonance (NMR) spectrum was recorded in DMSO-*d*_6_ on a Bruker brand spectrometer at 300 MHz. PerkinElmer brand DSC 4000 model instrument was used for differential scanning calorimetry measurement. As appropriate, pH measurements were performed using an AZ Instrument brand 86505 model pH meter (AZ Instrument Corporation, Taiwan). During the experimental process, a magnetic stirrer heater was used to prepare DES compounds and all heated agitation processes (DLAB brand MS-H280-Pro model, Istanbul/Türkiye).

### Preparation of standard solutions and deep eutectic solvents

Rhodamine B dye stock solution at a concentration of 1000 μg/mL was prepared with deionized water, and this stock solution was stored at 4 °C. Then, the standard solutions used in the study were prepared by diluting this stock solution with deionized water after determining the concentrations. A series of chemicals were tested to be used as HBA and HBD while preparing deep eutectic solvents. Thymol, decanoic acid, and TBAB components were utilized as HBA, while coumarin, menthol, and 1-octanol components were used as HBD. To perform selective extraction of Rhodamine B dye and to conduct experiments, the following DESs were prepared in different molar ratios: thymol-coumarin (2:1), decanoic acid-menthol (1:2), thymol-menthol (1:2), tetrabutylammonium bromide-1-octanol (1:2). After mixing HBA and HBD components with different molar ratios in a magnetic stirrer heater at 80 °C for half an hour, transparent, homogeneous, and fluid DES compounds were obtained.

### Optimization parameters

To determine Rhodamine B dye using the green and simple microextraction method based on deep eutectic solvent-assisted smartphone digital imaging colorimetry (GSME-DES-SDIC) method, DES type, molar ratio, volume, ultrasonic duration, pH, and centrifugation duration steps constituting each procedure step were optimized individually. In addition, optimization of many parameters such as the brightness of the light source, the position of the sample holder, the selection of an appropriate wavelength for the dye, and the decision of proper RGB channel was performed to analyze Rhodamine B dye precisely with the SDIC analysis method. The most suitable parameters were selected due to optimizations performed for both the procedure and analysis method, and the developed GSME-DES-SDIC procedure was applied to real samples.

### Real sample preparation

A comprehensive analysis of different products produced and frequently consumed by children was conducted. The materials analyzed in this study included sugar-coated snacks, cakes, fruit-flavored yogurt, herbal teas, and cotton candy. Various retail businesses supplied these products in Düzce. 2 g of cotton candy and colored dragees, fruit yogurt, and cake decorations; 9.3 mL of cherry-flavored drink; and 7 g of rosehip-flavored powdered drink were taken and dissolved in appropriate amounts of deionized water.

### GSME-DES-SDIC procedure

1 mL (1 µg mL^−1^) Rhodamine B dye, 300 μL DES, and 2 mL pH 4.00 buffer (acetic acid/sodium acetate) were added to a Falcon tube, respectively, and the total volume of the model solution was adjusted to 10 mL. The prepared solution was then subjected to an ultrasonic bath for 4 min to complete the extraction. Then, this solution was centrifuged at 4000 rpm for 3 min to ensure phase separation. The obtained DES phase was completed with methanol until the total volume reached 1 mL and transferred to a quartz micro cuvette. The quartz micro cuvette was placed in the prepared colorimetric box and analyzed with SDIC. Extraction efficiencies (EE) for each optimization parameter of the method developed in this study were calculated using the following Eq. 1 (Zhang, Wang, Guo, Nie, and Zhu, 2024).

1 $$\mathrm{EE}\;\left({\%}\right)=\frac{{\mathrm{C}}_{\mathrm{DES}}{\mathrm{V}}_{\mathrm{DES}}}{{\mathrm{C}}_{0}{\mathrm{V}}_{0}}\times\;100{\%}$$

**Fig. 1:**
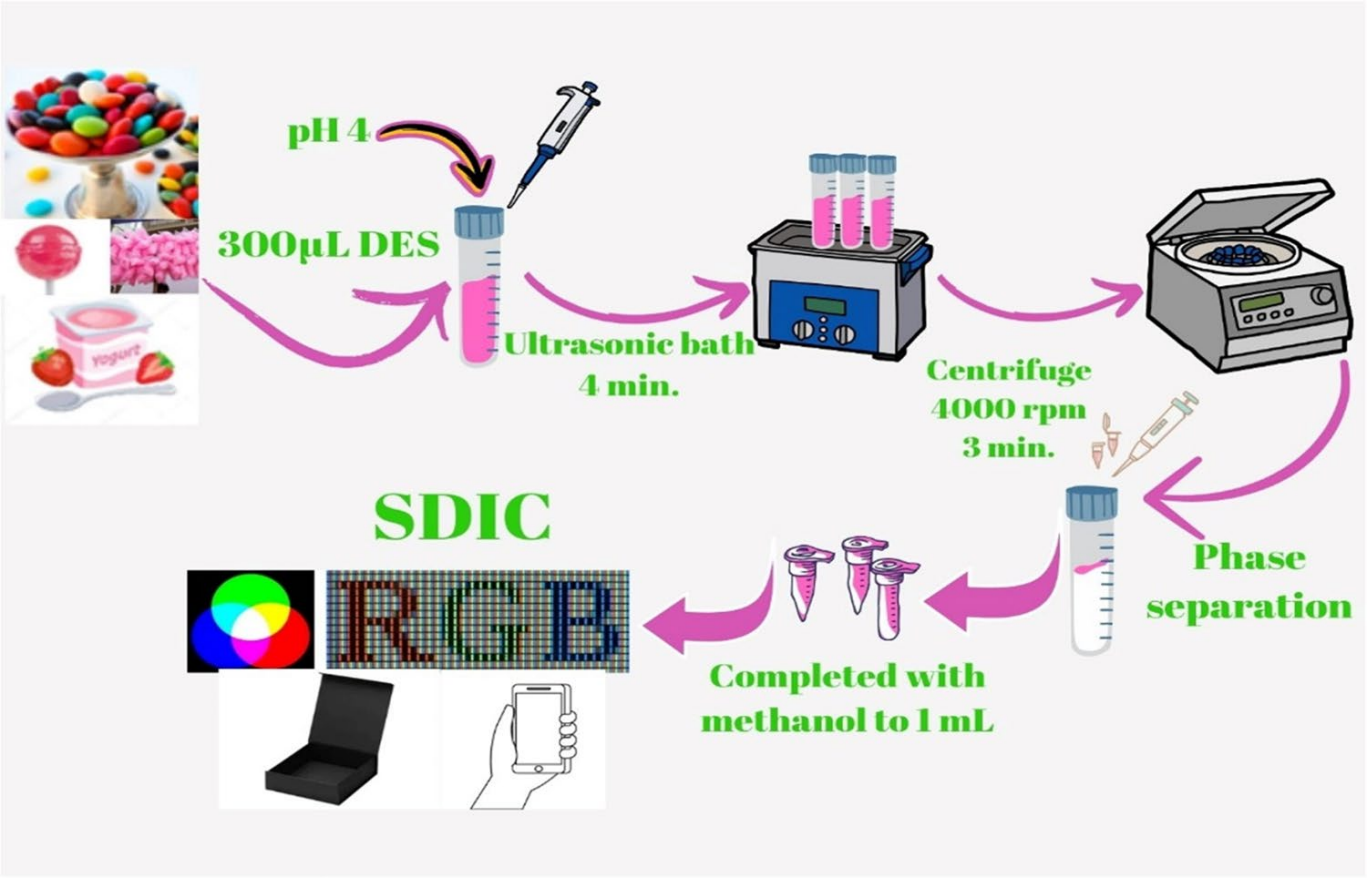
GSME-DES-SDIC procedure

Here, C_0_ and C_DES_ represent the analyte concentrations in the sample solution and diluted DES phase, respectively. V_0_ and V_DES_ represent the volume of the sample solution and the volume of the diluted DES phase, respectively. The developed GSME-DES-SDIC procedure is shown in Fig. 1.

### Data processing

The light from the source phone travels to the detector phone’s camera via the sample. The “RGB Color Detector” app on the detector phone measures the red, green, and blue color values of the light entering the detector. A color value of 255 means that the color is at its peak, whereas a color value of 0 means that the color is absent. Due to variations in light absorption from the source phone, RGB values change with sample concentration. As the solution concentration rises, the RGB values—the most significant color values—are in the blank data from the deionized water. Absorbance calculations use the color channel, which is particularly sensitive to changes in concentration between red, green, and blue colors. Since green light from the source phone had a wavelength of 554 nm, it was determined that the color value exhibiting the best absorbance in this study was red. In the blank solution, the green color value is seen as 255. Using the formula in Eq. 2, absorbance values were calculated based on the green color value [54].

2 $$A=- \mathrm{log}(I/{I}_{o})$$

In the equation, “A” represents the absorbance, and “I” represents the intensity of light passing through the solution, while “Io” represents the intensity of the incident light.

## Result and discussions

### Characterization of DES and mechanism

**Fig. 2:**
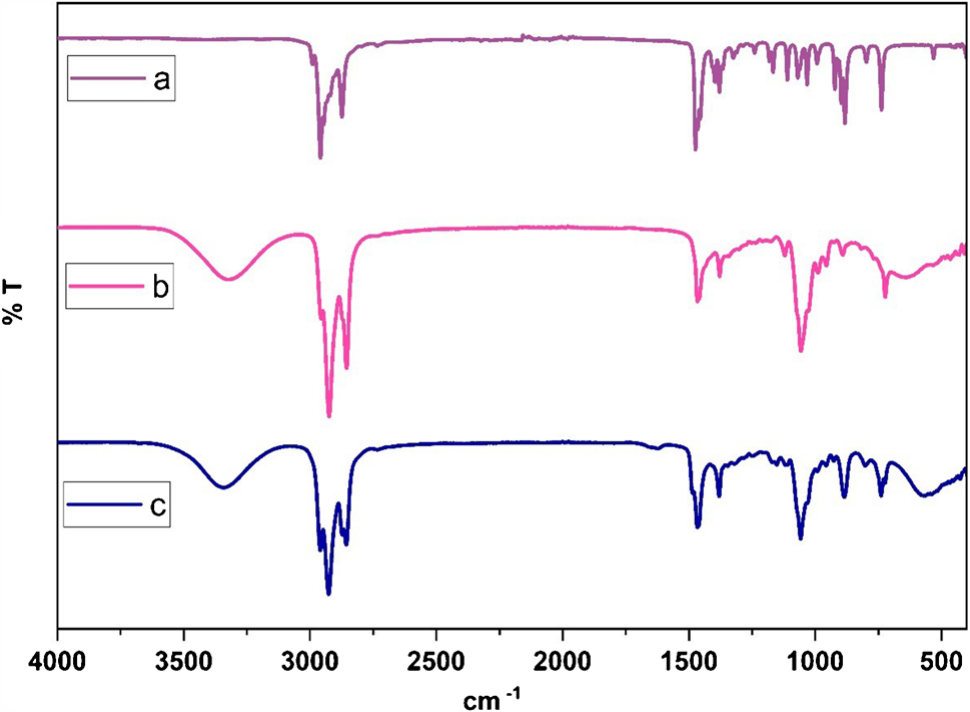
FT-IR spectra of **a** TBAB, **b** 1-OCL, and **c** DES

FT-IR spectra were recorded and interpreted to identify the functional and chemical structures of the DES compound and its constituent compounds TBAB and 1-octanol, and to explain the molecular interactions between them. Figure 2a–c show the FT-IR spectra of the DES compound, TBAB, and 1-octanol components. The FT-IR spectra of the 1-octanol and TBAB components and the DES compound in the 4000–400 cm^−1^ range clearly show the characteristic peak vibrations. The peaks at 2958 and 2873 cm^−1^ in the FT-IR spectrum of the pure TBAB compound correspond to C-H stretching vibrations, while the C-N stretching vibration is observed at 1031 and 1166 cm^−1^ [55, 56]. In the FT-IR spectrum of 1-octanol, the bands and peaks at 3326, 2924, 2855, 1465, 1378, 1056, and 890 cm^−1^ correspond to the stretching vibrations of O-H groups, symmetric C-H stretching vibrations, antisymmetric bending of C-H bonds in the structure of CH_3_ groups, symmetric bending of C-H bonds in the structure of the CH_3_ group, stretching vibrations of C-O bonds, and stretching vibrations of C-C and C-O bonds together. Looking at the FT-IR spectrum of compound DES, although the characteristic peaks belonging to 1-octanol and TBAB compounds were observed, the vibration at 3326 cm^−1^ of the OH group in the structure of the 1-octanol compound shifted to 3339 cm^−1^ in the DES compound, proving the hydrogen bond formation between the components. Furthermore, the FT-IR spectrum of the DES compound shows vibrations at 1107, 2856, and 2926 cm^−1^ corresponding to the C-O bending peak and C-H stretching in the structure of CH_2_ and CH_3_, respectively [57–59].

**Fig. 3:**
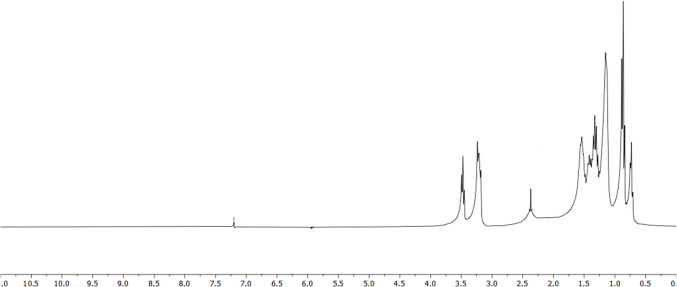
NMR spectrum of the DES

The ^1^H-NMR spectrum of the DES compound is given in Fig. 3. When the ^1^H-NMR spectrum of the DES compound obtained from TBAB and 1-octanol was examined, it was seen that ~δ 1.22, 1.42, 2.30, and 3.47 signals were obtained, and the peak observed at 2.30 ppm shows the presence of hydrogen bonding between HBD and HBA compounds. Since the structures of TBAB and 1-octanol, which form DES, consist of long-chain alkyls, overlapping occurs in the proton signals in the ^1^H-NMR spectrum and cannot be integrated separately [60, 61].

**Fig. 4:**
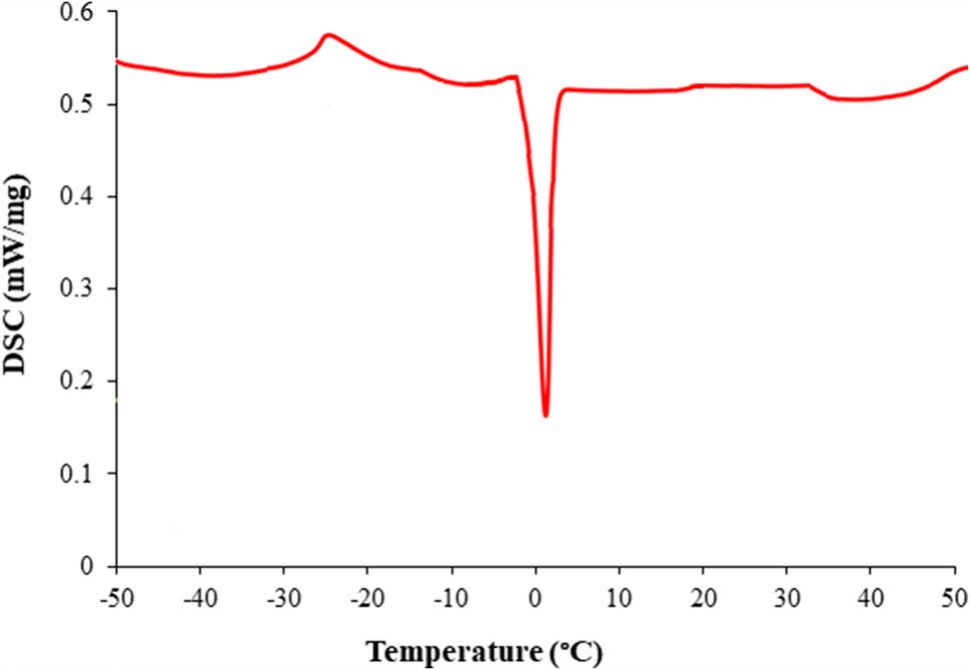
DSC thermogram of the DES

Differential scanning calorimetry (DSC) analysis of the synthesized DES was conducted at a rate of 5 °C/min from −50 °C to 50 °C. The DSC thermogram of the DES compound is given in Fig. 4. The DES compound showed cold crystallization below −25 °C, then melting at 1 °C.

The characterization techniques mentioned above explained and demonstrated the genesis of DES. The mechanism of the developed method is thought to be as follows. The formation theory of DESs states that 1-octanol and the halide anion of DES form hydrogen bonds. The dissociation of DES was thought to be associated with the dissolution of TBAB in the aqueous phase, whereas 1-octanol is in charge of the production of the dispersed organic phase. Thus, the dissociation of TBAB in the aqueous sample phase and the dissociation of DES due to octanol hydration were linked to the extraction mechanism. According to this perspective, TBAB can be seen as a dispersive agent, similar to how organic phase dispersion is achieved in microextraction investigations using dispersive solvent. TBAB can also function as a desalting agent and facilitate mass transfer between the two phases because it is a quaternary ammonium salt that contains the bromide ion [60].

### Optimization of SDIC conditions

#### Construction of the colorimetric box

**Fig. 5:**
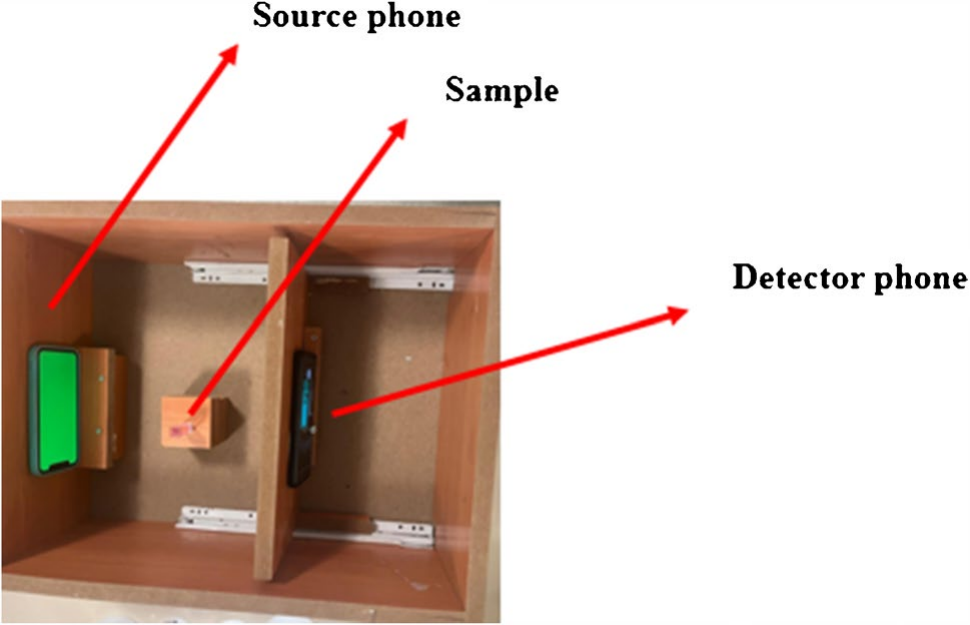
Colorimetric box

A two-compartment box with a black interior was set up for colorimetric measurements. An iPhone 6 smartphone (source phone) was positioned 8 cm from a sample holder, which was placed in the middle of the first section as a light source. A Samsung Galaxy S21 FE 5G smartphone was positioned as a detector in the second compartment. There is an adjustable distance between the sample and the detector phone. The “RGB Color Detector” app was set up for RGB measurements on the detector phone. Figure 5 shows the construction of the colorimetric box [62].

#### Selection of the RGB channel and the detection wavelength

**Fig. 6:**
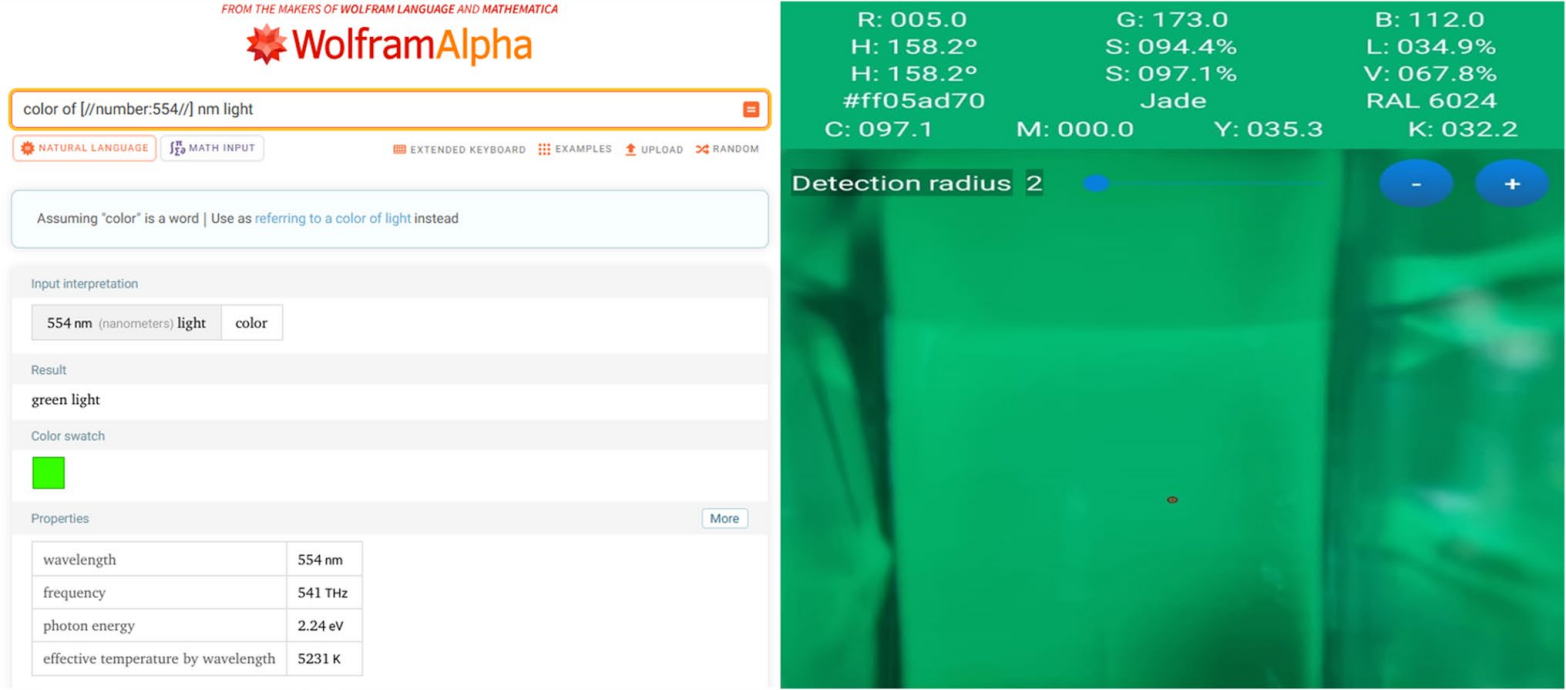
The wavelength of the color and screenshot of the application

The RGB image comprises three channels: red, green, and blue. Absorbance is employed to select the RGB channel, and the channel that yields the optimal absorbance is utilized in the study. The G (green) channel was chosen due to its superior absorbance values. In the application used on the phone, the G (green) channel exhibited a value of 255 at the selected wavelength. This value represents the maximum possible for the blank sample. Subsequently, the transition from absorption to concentration was calculated using the established formula. Instead of using continuous light sources to illuminate the sample within the colorimetric box, the employment of monochromatic light sources, which possess enhanced sensitivity and selectivity, has emerged as the prevailing preference. Given these considerations, a monochromatic light source with an analyte-specific color was utilized, with the light source positioned on the smartphone screen. The color corresponding to the wavelength was identified using Wolfram Alpha (https://www.wolframalpha.com). The wavelength was obtained at 554 nm, the maximum response indicated in the application. The image taken from the WolframAlpha website and the screenshot taken from the RGB Color Detector application for selecting the color to be worked on via wavelength are given in Fig. 6.

#### Position of the sample holder

**Fig. 7:**
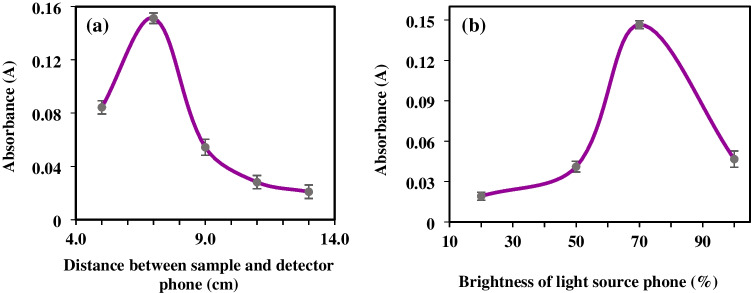
Selection of **a** distance of the sample to the detector and **b** brightness of the light source

A black wooden box with two compartments was designed and constructed in the laboratory environment. A sample of orange was then placed in the center of the phones, which were used as both the detector and the beam source. The sample was placed in a quartz microcuvette, and the distance between the sample and the detector was examined to determine the difference between the results. Initial trials, encompassing five trials between 5 and 13 cm, revealed a blue shift in the images captured at 5 cm. However, images obtained at 9, 11, and 13 cm could not be read due to stray rays from the bathtub and focusing issues. Consequently, a distance of 7 cm was determined as the acceptable range for measurement. The ideal value, as seen in Fig. 7a, was 7 cm.

#### Brightness of the light source

The brightness of the light source is a parameter that significantly affects absorption. Measurements were taken at 20, 50, 70, and 100% screen brightness to ascertain this relationship. It was also observed that the absorbance decreased at very high brightness; at very low brightness, the G (green) value dropped below 255. As a result of the measurements, the brightness used in the study was determined to be 70%. The most suitable light source to be used in the study was selected as 70% and is shown in Fig. 7b.

### Optimization of GSME-DES-SDIC conditions

#### Determination of the type, molar ratio, and volume of DES

**Fig. 8:**
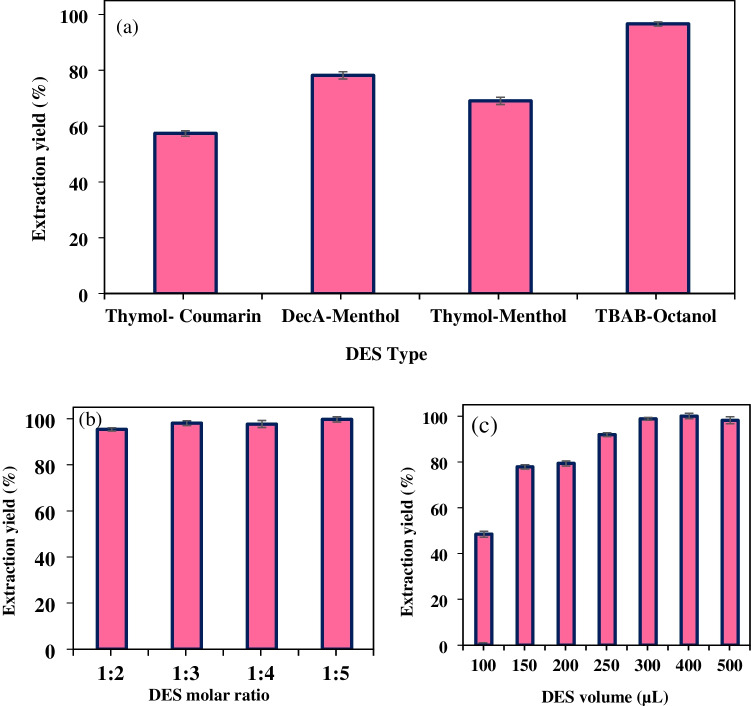
Effect of the **a** type, **b** molar ratio, and **c** volume of DES

DESs are naturally occurring, flammable, volatile, and non-toxic environmentally friendly solvents. They are also easy to synthesize, cost-effective, and easily recycled after use. The chemical properties of these solvents, which have high extraction efficiency and similar physical properties to ionic liquids, are different from ionic liquids [63]. One of its components is a hydrogen bond acceptor (HBA), and the other is a hydrogen bond donor (HBD). Determining the type of DES, the ratios of this type, and the volume of DES obtained is a critical step in terms of extraction efficiency. In the study, four different DES types consisting of thymol: coumarin, decanoic acid: menthol, thymol: menthol, and tetrabutylammonium bromide: octanol were prepared in various molar ratios, and Rhodamine B was determined with the developed method. After the analyses, the highest extraction efficiency was observed when tetrabutylammonium bromide and octanol were used (Fig. 8a). When the effect of extraction efficiency on DES molar ratio was examined, the developed method prepared and applied molar ratios of tetrabutylammonium bromide and octanol varying from 1:2 to 1:5. In this step, it was observed that DES prepared with a 1:2 molar ratio of tetrabutylammonium bromide and octanol had a good extraction efficiency with Rhodamine B (Fig. 8b). At higher molar ratios (e.g., 1:5), the extraction efficiency did not change, so the use of a smaller amount of octanol was preferred due to both the amount of substance and the energy savings in the preparation phase, so a 1:2 molar ratio of tetrabutylammonium bromide and octanol was used in the remainder of the study. A volumetric study was conducted on the determined DES, with trials ranging from 100 to 500 µL. The outcome of this trial indicated that 300 µL would be the optimal DES volume, as it was observed to yield the highest extraction yield and did not demonstrate any change in subsequent trials (Fig. 8c).

#### Determination of solution pH

**Fig. 9:**
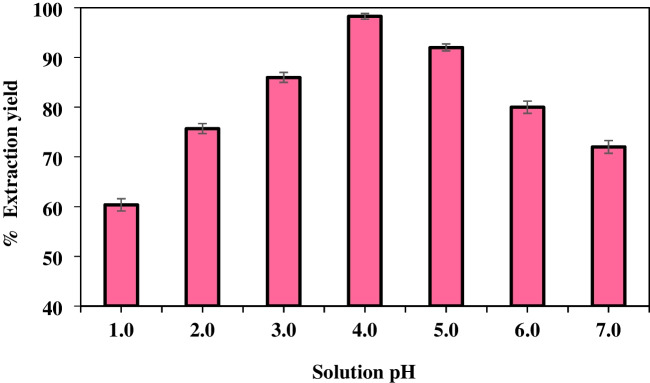
Effect of solution pH

In extraction processes, sample pH is a critical parameter that must be meticulously monitored [64]. The pH of the sample solution was analyzed within the range of 1.00–7.00. It was observed that the highest extraction efficiency was achieved at pH 4, thus establishing this as the optimal pH for the procedure. Subsequent trials conducted at pH 4.00 and above revealed deviations in the RGB values attributable to alterations in the color of the solution. Rhodamine B dye shows improved stability and coloration under acidic pH conditions. In contrast, changes in the chemical structure of the dye can occur in basic environments. In particular, the basic structure of Rhodamine B dye may undergo deprotonation at high pH levels, resulting in changes in color or decreased solubility properties. Given these limitations, it is generally recommended to maintain neutral or acidic pH values during studies involving Rhodamine B dye. In our study, we deliberately avoided working in the base region based on extensive literature research to eliminate these potential drawbacks (Fig. 9).

#### Ultrasonic and centrifuge time

**Fig. 10:**
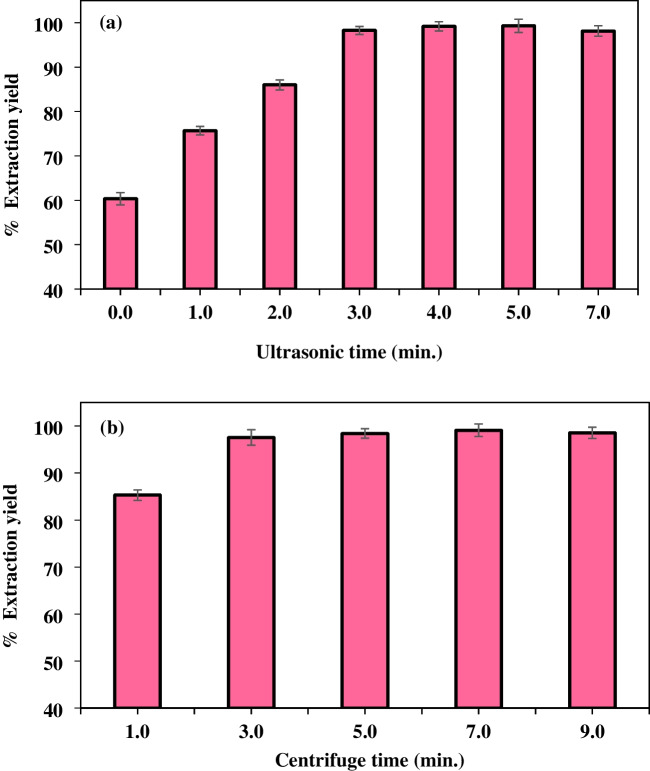
Effect of **a** ultrasonic and **b** centrifuge time

In green chemistry applications, time and practicality are critical issues. In liquid-phase microextraction methods, one of the ways used to both increase extraction efficiency and reduce extraction time is the mixing time. The mixing step is vital for more efficient and rapid separation of the organic extraction solvent phase from the aqueous phase. Ultrasonication is an integral component in the homogenization of sample solutions and facilitates the removal of contaminants by heat application. In this study, measurements were made to observe the effects of ultrasonication on the recovery of Rhodamine B without any ultrasonication and at a time interval of 7.0 min. The most suitable extraction efficiency was obtained at 4.0 min after ultrasonication (Fig. 10a). Therefore, the optimum mixing time of the study was 4.0 min.

It is known that the quality of phase separation significantly affects the results of extraction processes. For this reason, centrifugation was applied at 4000 rpm for 1.0–9.0 min to ensure the separation of phases. When the results of the extraction efficiencies obtained are examined in Fig. 10b, it is seen that the centrifugation time of 3.0 min is sufficient, and there is no contribution to the extraction efficiency after the 3rd minute. As a result, the optimum time for a good phase separation observation and a short analysis time was determined as 3.0 min, and studies were carried out by taking these values into account in the following study.

#### Sample volume

**Fig. 11:**
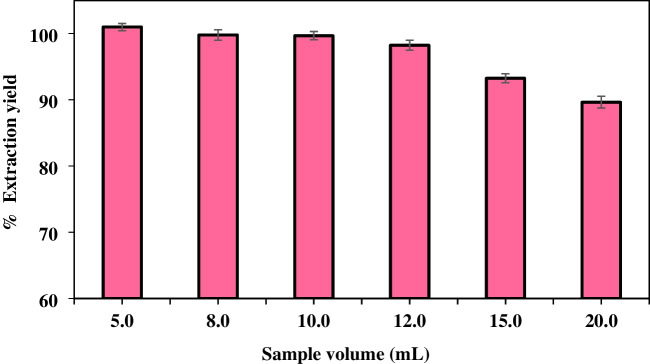
Effect of sample volume

The sample volume constitutes a pivotal component in the overall efficacy of the extraction process. Elevated sample volumes have resulted in diminished extraction efficiency, attributable to the over-dissolution of the extraction solvent. Conversely, augmented sample volumes have been shown to enhance extraction efficiency by increasing the quantity of the target analyte, thereby improving the sensitivity of the analytical method [65, 66]. The effect of sample volume on the recovery of the sample volume in the extraction from the aqueous phase to the DES phase was investigated in the 5.0–20.0 mL range. Studies (Fig. 11) show that recoveries are low at 15 mL and above and decrease as sample volume increases. Therefore, 12 mL was selected as the optimum sample volume. Since the sample volume of the developed method is 12.0 mL and the DES volume is 300 µL, the enrichment factor was found to be 40.

#### Effect of matrix ions/dyes

The results obtained by examining the effects of matrix ions and dyes on the extraction recoveries of Rhodamine B dye for the developed SDIC method are given in Table 1. For this reason, some anions, cations, and dyes were added to the model test solution with Rhodamine B dye at various concentrations, and their potential effects were investigated. It was determined that there was no negative effect on the determination of Rhodamine B dye in the presence of interfering species at the concentrations summarized in Table 1. When the results were examined, it was seen that the extraction recoveries of Rhodamine B dye in the presence of dyes and matrix ions were between 93 and 99%. This shows that the method works with high accuracy and selectivity in the presence of interfering species.

**Table 1:** Effect of matrix ions/dyes

Ions/dyes	Concentrations (µg/mL)	Recovery (%)
K^+^	500	97±3
Na^+^	500	95±3
Mg^2+^	100	94±4
Ca^2+^	100	98±1
Al^3+^	100	95±3
NO_3_^−^	100	99±2
SO_4_^2−^	100	97±3
PO_4_^3−^	100	95±4
Sudan dye IV	10	98±2
Ponceau 4R	10	93±4
Tartrazine	10	96±3
Brilliant Blue FCF	10	94±2
Allura Red	10	99±1

### Method analytical performance

The analytical parameters, such as linear range, detection limit (LOD), and quantification limit (LOQ) of the method developed for determining Rhodamine B dye with SDIC, were determined by applying the method to the prepared model solutions under optimum conditions. The linear working range of the method was determined by applying the developed method to standard solutions containing Rhodamine B at six different concentrations in the range of 0.01–5.0 μg/mL. The calibration correct equation of the method was found as A = 0.3131 C + 0.1466 with a regression coefficient (R^2^) of 0.9996. The regression equation shows that the concentration in the range studied is C (μg/mL)/absorbance (A). LOD and LOQ values were determined as 0.0054 μg/mL and 0.018 μg/mL, respectively, due to readings made at 554 nm with seven parallel blank solutions. While the equation (3SD/m) was used for LOD in the calculations, the equation (10SD/m) was considered for LOQ. Here, ss is the standard deviation of the concentrations of blank samples, and m is the slope of the calibration line. Since the analyte was extracted from 12 mL to 300 μL DES, the enrichment factor was 40. In addition, 11 parallel model solutions containing 50 μg/L Rhodamine B were prepared, and the method’s precision was determined by determining the % relative standard deviation (% RSD). Accordingly, the %RSD (SD/X_avg_×100) value of the developed method was 1.29% and 2.03% intraday and interday, respectively. Here, SD is the standard deviation of parallel readings, and Xort is the average value.

### Determination of Rhodamine B in food samples

After optimization studies (optimum microextraction conditions: pH: 4; DES: TBAB: OCL (1:2); DES volume: 300 μL; ultrasonication time: 4 min; centrifugation time: 4000 rpm 3 min; analysis at 554 nm with SDIC), addition-recovery studies were performed on pink-colored colorful dragee, cherry-flavored drink, cake decoration, fruit yogurt, rosehip-flavored powder drink, and cotton candy samples taken from Düzce province markets to determine the accuracy of the method. For this purpose, the developed microextraction method was applied under optimum conditions by preparing three parallel samples for each sample. The analysis results obtained are given in Table 2. When the results were examined, the recovery values obtained from the addition-recovery were quantitative (≥95%). The recovery values obtained in the determination of Rhodamine B dye with the GSME-DES-SDIC method developed in this study show that the method has high accuracy.

**Table 2:** Addition-recovery study for different food samples (*N*=3)

Sample	Found in real sample (μg mL^−1^)	Added (μg mL^−1)^	Found (μg mL^−1^)	R (%)
Colorful dragee	-	-	-	-
		0.5	0.52	103.88
		2.5	2.42	96.93
Cherry-flavored drink	-	-	-	-
		0.5	0.49	97.87
		2.5	2.43	97.06
Cake decoration	8.24	-	-	-
		0.5	8.79	100.61
		2.5	10.22	95.16
Fruit yogurt	-	-	-	-
		0.5	0.51	101.80
		2.5	2.55	101.93
Rosehip-flavored powder drink	36.06	-	-	-
		0.5	34.98	95.69
		2.5	37.48	97.21
Cotton candy	4.44	-	-	-
		0.5	4.82	97.66
		2.5	6.76	97.47

### Comparison with other methods

When comparing the GSME-DES-SDIC procedure developed in this study with other studies in the literature, it is seen that better results are obtained. The comparative results of the GSME-DES-SDIC procedure developed in this study with the enrichment methods in the literature are given in Table 3. When evaluated in terms of correlation coefficient and detection limit, it is seen that the developed method can be compared with the SPE, DES-LPME, and DES methods in the literature, and the obtained values are much better. In addition, the fact that the innovative method in this study gives competitive results with the studies in the literature is promising for other studies to be developed.

**Table 3:** Comparison of the GSME-DES-SDIC method with the literature

Sample	Method	LOD (μgL^-1^)	LDR (μg mL^-1^)	RSD (%)	PF	Ref.
Foodstuff and industrial samples	VA-DES-SLPME	13	0.042–3.0	6.0	40	[67]
Food and water samples	SA-LPME-QH-DES	8	0.029–0.4	3.6	10	[68]
Candy, water, plastic, cosmetic	VA-MSPE	0.83	0.83–2.77	3.8	71.4	[69]
Food samples	GSME-DES-SDIC	5.4	0.01–5.0	1.29	40	This study

*VA-DES-SLPME, *vortex assisted deep eutectic solvent solid liquid phase microextraction;* SA-LPME-QH-DES, *sonication-assisted liquid-phase microextraction-quasi-hydrophobic deep eutectic solvents;* VA-MSPE,* vortex assisted magnetic solid phase extraction method; *GSME-DES-SDIC*, green and simple microextraction method based on deep eutectic solvent-assisted smartphone digital image colorimetry

## Conclusion

A significant research has been conducted to analyze Rhodamine B dye in various food and beverage samples using a deep eutectic solvent, commonly referred to as “green solvent.” The developed method for the microextraction and determination of Rhodamine B dye used the DES compound consisting of TBAB and 1-octanol. In the Rhodamine B dyestuff microextraction studies, TBAB is used as a dispersant, and 1-octanol is used as an extraction solvent. In addition to its use as a dispersant, TBAB is thought to provide mass transfer between organic phases. In light of the rapid technological advances and the urgent need for efficiency, a new method consisting of SDIC and deep eutectic solvent has been developed to complement the current research. The present study adopts the principle of a traditional method, UV/Vis, with the help of a detection camera and sample holders placed in a colorimetric wooden box prepared in a laboratory environment. SDIC, an efficient analysis method, is quite innovative in that it reduces the need for complex, inaccessible, and expensive instrumentation. It reduces the cost, unlike traditional methods, by using smartphone cameras. In addition, the rapid applicability and portability of the SDIC method make it suitable for on-site applications. Following a series of analyses, phase separation of Rhodamine B staining was found to be highly effective, with the analysis concluded by employing TBAB:OCL DES at a ratio of 1:2, which was observed to yield optimal results. The outcomes of this study substantiate the precision and reliability of the method. Notably, the study is characterized by its expeditiousness, cost-effectiveness, and environmental sustainability, attributable to using environmentally friendly solvents. Considering the results obtained, the SDIC procedure combined with DES for the determination of Rhodamine B dye will be a pioneering study for many similar future studies, with its feature of improving existing analysis methods and its sustainability aspect.

## Data Availability

The authors confirm that the data supporting the findings of this study are available within the article.
